# Supplementary material for: Wheat Elongator Subunit 4 Negatively Regulates Freezing Tolerance by Regulating Ethylene Accumulation
Source: Int J Mol Sci. 2022 Jul 11;23(14):7634. doi: 10.3390/ijms23147634 (PMC9324374; doi:10.3390/ijms23147634)
Supplement: Supplementary file 1 [file ijms-23-07634-s001.zip › Table-S1.pdf]

Table S1 – Primers used and their sequences.

Primer name	5'-3'	usage
TaELP4-7B-F	ATGGCTGCACCAAACGTTCG	Amplification of full-length cds of TaELP4-7B
TaELP4-7B-R	TTAGAAATCGAGCTGCGAGC	
VIGS-TaELP4-F	AACGCTAGCTGGACGAGCAAGTGATGAGG	Construction of vector γ-TaELP4
VIGS-TaELP4-R	GGCGCTAGCCTGCACTAAGATTTCCACCCTC	
16318h-TaELP4-7B-GFP-F	GCTGTACAAGGTCGACATGGCCGCCGCCGCCG	Construction of subcellur localization vector 35S::TaELP4-7B-GFP
16318h-TaELP4-7B-GFP-R	TACCGGATCCACTAGTGAAATCGAGCTGCGAG	
16318h-AtELP4-GFP-F	AGGACAGCCCAAGCTTATGGCTGCACCAAACGTTCG	Construction of subcellur localization vector 35S::AtELP4-GFP
16318h-AtELP4-GFP-R	GCAGGCATGCAAGCTTAAAATCTAGTGCTCCGGAT	
1300nGFP-8530-inF	GCTGTACAAGGTCGACATGGCCGCCGCCGCCG	Construction of transgenetic vector 35S:TaELP4-7B-GFP
1300nGFP-8530-inR	TACCGGATCCACTAGTGAAATCGAGCTGCGAG	
qTaELP4-7A-F	CTCTTCGCTGCTCCCATGAA	Primers for RT-qPCR
qTaELP4-7A-R	TGCCAAGCTATCCTCAAGCC	
qTaELP4-7B-F	GCTCAGAAAGGCTCCTGGTT	
qTaELP4-7B-R	TGGGATGGACAAGGCTTAGC	
qTaELP4-7D-F	GCTCGGTCGTGATGGTCAT	
qTaELP4-7D-R	CACCGGAATTCCTCTCGTCC	
BSMV-CP-F	TGACTGCTAAGGGTGGAGGA	
BSMV-CP-R	CGGTTGAACATCACGA AGAGT	
qTaACS2-F	CTCTTCTCGTGGATGGACCT	
qTaACS2-R	ATGGTCTCGTCGTCCATGTT	
qTaACS6-F	GATTTGGTACGTACGCACTTTT	
qTaACS6-R	CTAAACCTTCCCAAACAATCGC	
qTaActin-F	GGAATCCATGAGACCACCTAC	
qTaActin-R	GACCCAGACAACTCGCAAC	
qAtEIN2-F	CGCAAGCATCGTCCCTCACAATTT	
qAtEIN2-R	ACAAGTGACAGTCCGCTGAAGACA	
qAtEIN3-F	TCCTGCACCTTACAAGAAGCCTCA	
qAtEIN3-R	TTGCCTCACGAGCTTACGGATCTT	
qAtEIL1-F	AGCTTCAGGACACGACTCTTGGTT	
qAtEIL1-R	TTAGGCCACCAAGGTGGAGAAACT	
qAtARR5-F	TGATCGGAAGTTCATCGAGCGGTT	
qAtARR5-R	TCCCAGGCATAGAGTAATCCGTCA	

qAtCBF1-F	GCATGTCTCAACTTCGCTGA	
qAtCBF1-R	ATCGTCTCCTCCATGTCCAG	
qAtCBF2-F	TGACGTGTCCTTATGGAGCTA	
qAtCBF2-R	CTGCACTCAAAAACATTTGCA	
qAtCBF3-F	GATGACGACGTATCGTTATGGA	
qAtCBF3-R	ACACTCGTTTCTCAGTTTTACAAAC	
qAtRD29A-F	GCCGAGAAACTTCAGATTGG	
qAtRD29A-R	CCATTCCTCCTCCTCCTTTC	
qAtCOR15b-F	AAAGCAGAGTGGTGTTGGTACCGT	
qAtCOR15b-R	TCATCGAGGATGTTGCCGTCACTT	
qAtKIN1-F	ACCAACAAGAATGCCTTCCA	
qAtKIN1-R	CCGCATCCGATACACTCTTT	
qAtCOR47-F	CAGTGTCGGAGAGTGTGGTG	
qAtCOR47-R	ACAGCTGGTGAATCCTCTGC	
qAtActin-F	GGTAACATTGTGCTCAGTGGTGG	
qAtActin-R	AACGACCTTAATCTTCATGCTGC	
qAtACS2-F	AGGAAGAACATTCACCTAGTCG	
qAtACS2-R	CTCTAAAACCAGGAAGTCCCAT	
qAtACS6-F	CTGAATTCAGACAAGCTGTAGC	
qAtACS6-R	CGGGATTAGCTAAACAGAAAGC	
qAtACS2-156F	TTTGGTCTCCTCAAGGTTTCTG	
qAtACS2+8R	AGACCCATTTGCTGTGTCAAT	
qAtACS2+784F	GAGAGGGTGGTTATGAGCG	
qAtACS2+884R	AGTACGGGGAGGGAATGAG	
qAtACS2+1386F	AGGAAGAACATTCACCTAGTCG	
qAtACS2+1561R	CTCTAAAACCAGGAAGTCCCAT	
qAtACS2+1966F	ATACTCTCCATGTGGCGCTTG	
qAtACS2+2152R	TGGAGACGAGAGACCATCCT	Primers for ChIP-qPCR
qAtACS6-325F	TCTCAGGTCCCCCACAATGA	
qAtACS6-126R	ACCGAGTATGTGGAGCGTTG	
qAtACS6+42F	TTGTCTAAAATCGCCTCCGGT	
qAtACS6+290R	ACGCATCAAATCTCCACAAAGC	
qAtACS6+583F	CACACGAGACGGTTGCTTTC	
qAtACS6+774R	CACTCCGGTTCTCCATCTCA	
qAtACS6+1598F	ACCGATGGCTGCAACAACTAT	
qAtACS6+1713R	ATGAGGCGAGAAGAAGCCATC	
