# Supplementary figures and images for: Wheat Elongator Subunit 4 Negatively Regulates Freezing Tolerance by Regulating Ethylene Accumulation
Source: Int J Mol Sci. 2022 Jul 11;23(14):7634. doi: 10.3390/ijms23147634 (PMC9324374; doi:10.3390/ijms23147634)

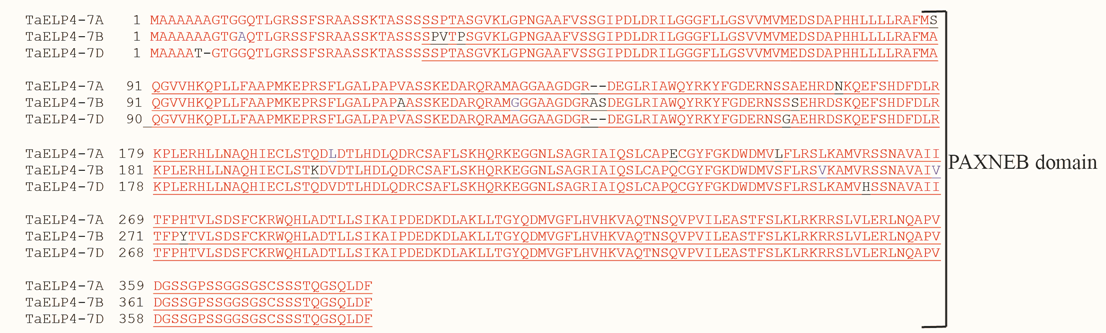

Supplement: Supplementary file 1 [file ijms-23-07634-s001.zip › Figure-S1.tif]

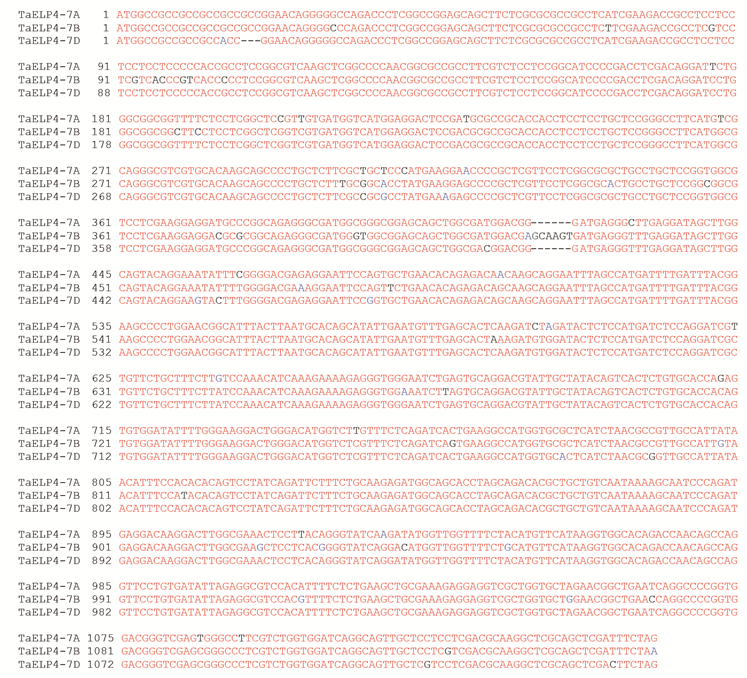

Supplement: Supplementary file 1 [file ijms-23-07634-s001.zip › Figure-S2.tif]

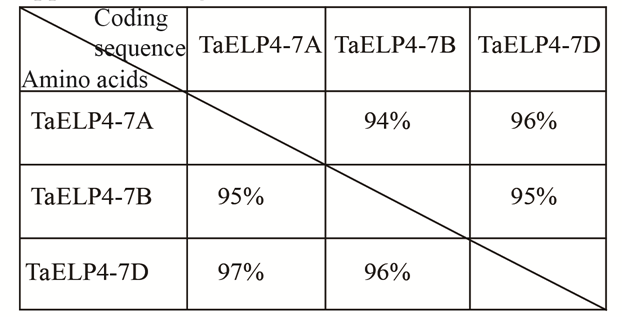

Supplement: Supplementary file 1 [file ijms-23-07634-s001.zip › Figure-S3.tif]

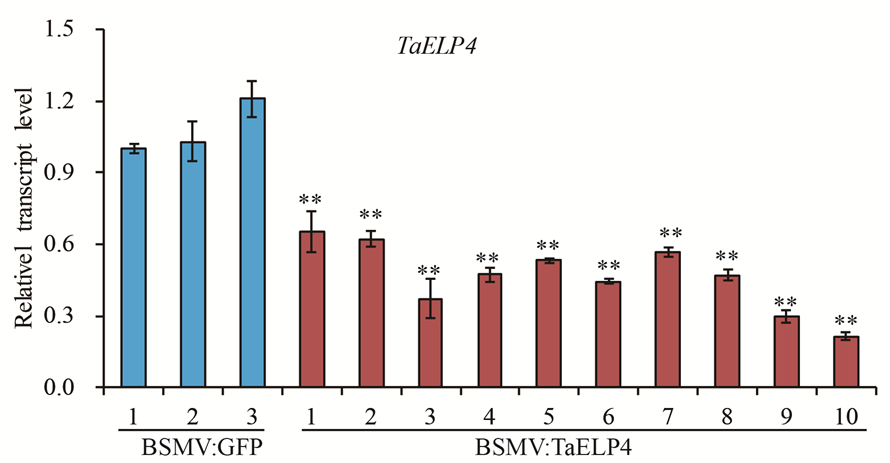

Supplement: Supplementary file 1 [file ijms-23-07634-s001.zip › Figure-S4.tif]

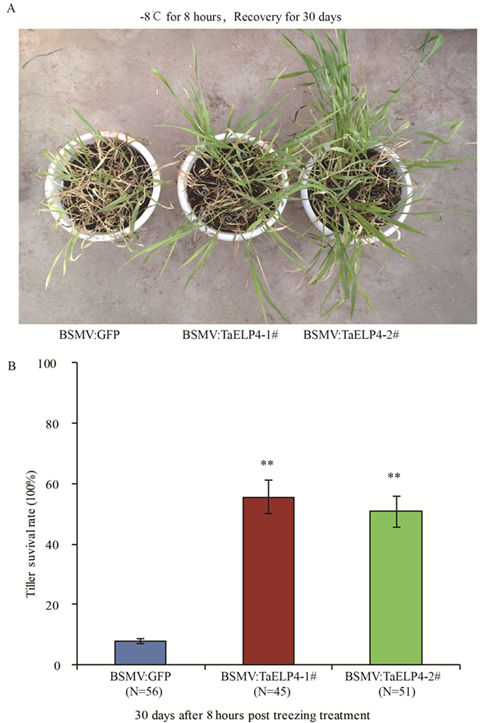

Supplement: Supplementary file 1 [file ijms-23-07634-s001.zip › Figure-S5.tif]

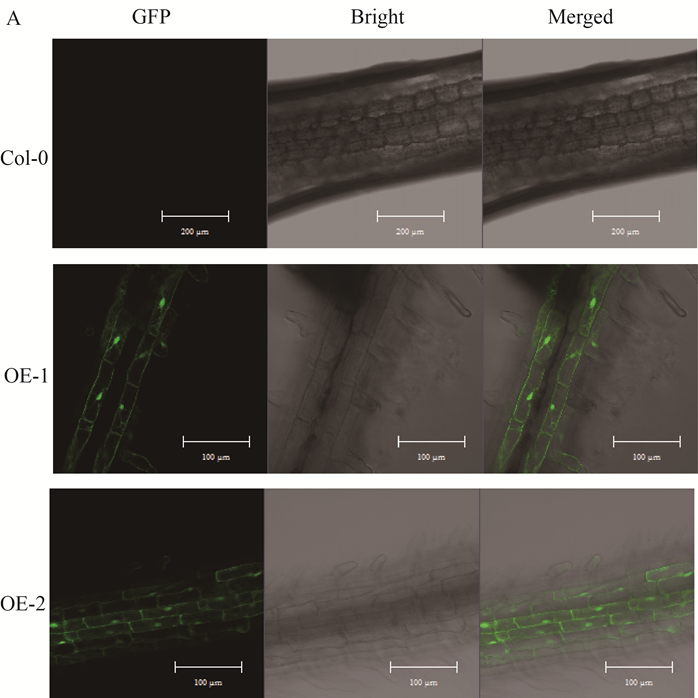

Supplement: Supplementary file 1 [file ijms-23-07634-s001.zip › Figure-S6.tif]

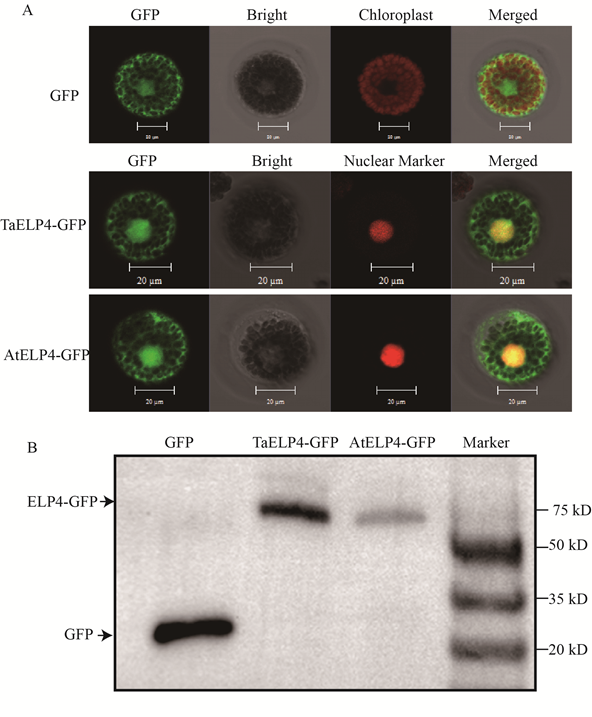

Supplement: Supplementary file 1 [file ijms-23-07634-s001.zip › Figure-S7.tif]
